# Herbicide tolerance-assisted multiplex targeted nucleotide substitution in rice

**DOI:** 10.1016/j.dib.2018.08.124

**Published:** 2018-08-30

**Authors:** Zenpei Shimatani, Ushio Fujikura, Hisaki Ishii, Rie Terada, Keiji Nishida, Akihiko Kondo

**Affiliations:** a Graduate School of Science, Technology and Innovation, Kobe University, 1-1 Rokkodai-cho, Nada-ku, Kobe, Hyogo 657-8501, Japan; b Graduate School of Agriculture, Meijo University, 1-501 Shiogamaguchi, Tempaku-ku, Nagoya, Aichi 468-8502, Japan; c Department of Chemical Science and Engineering, Graduate School of Engineering, Kobe University, 1-1 Rokkodai-cho, Nada-ku, Kobe, Hyogo 657-8501, Japan

## Abstract

Acetolactate synthase (ALS) catalyzes the initial step in the biosynthesis of branched-chain amino acids, and is highly conserved from bacteria to higher plants. ALS is encoded by a single copy gene in rice genome and is a target enzyme of several classes of herbicides. Although *ALS* mutations conferring herbicide-resistance property to plants are well documented, effect of Imazamox (IMZ) on rice and the mutations in *ALS* correlated with IMZ tolerance were unclear. In this article, the effect of IMZ on rice calli and seedlings in tissue culture conditions were evaluated. Also, the *ALSA96V* mutation was confirmed to improve IMZ tolerance of rice calli. Based on these results, ALS-assisted multiplex targeted base editing in rice was demonstrated in combination with Target-AID, a CRISPR/Cas9-cytidine deaminase fusion system [1], [2].

**Specifications table**

**Table:** 

Subject area	Biotechnology
More specific subject area	Plant Biotechnology, Plant genome editing and Plant breeding technique
Type of data	Tables and Figures
How data was acquired	Agrobacterium-mediated transformation, Plant tissue culture technique, Sanger sequencing and Fluorescence microscopic analysis
Data format	Analyzed data
Experimental factors	Not applicable
Experimental features	Multiplex targeted base editing using the Target-AID system [1] and rice callus lines carrying *switch-mEGFP*[2]
Data source location	Kobe University, Kobe, Japan
Data accessibility	Data are provided with this article
Related research article	Z. Shimatani, U. Fujikura, H. Ishii, Y. Matsui, M. Suzuki, Y. Ueke, K. Taoka, R. Terada, K. Nishida and A. Kondo Inheritance of co-edited genes by CRISPR-based targeted nucleotide substitutions in rice. Plant Physiol Biochem. 131 (2018), pp. 78-83.

**Value of the data**

● Effective IMZ concentrations were determined to suppress rice callus proliferation and seedling growth in tissue culture conditions.
● The data demonstrated that *ALS A96V* mutation confers IMZ tolerance to rice calli and is thus it is applicable as an endogenous selectable marker indicating the activity of Target-AID system.
● Simultaneous engineering of multiplex traits of rice calli was successfully demonstrated by Target-AID in combination with ALS-assisted selection. This will contribute to more efficient selection of the prospective cells carrying desired mutations as IMZ tolerance provide a useful index of Target-AID activity.

## 1 Data

**Fig. 1:**
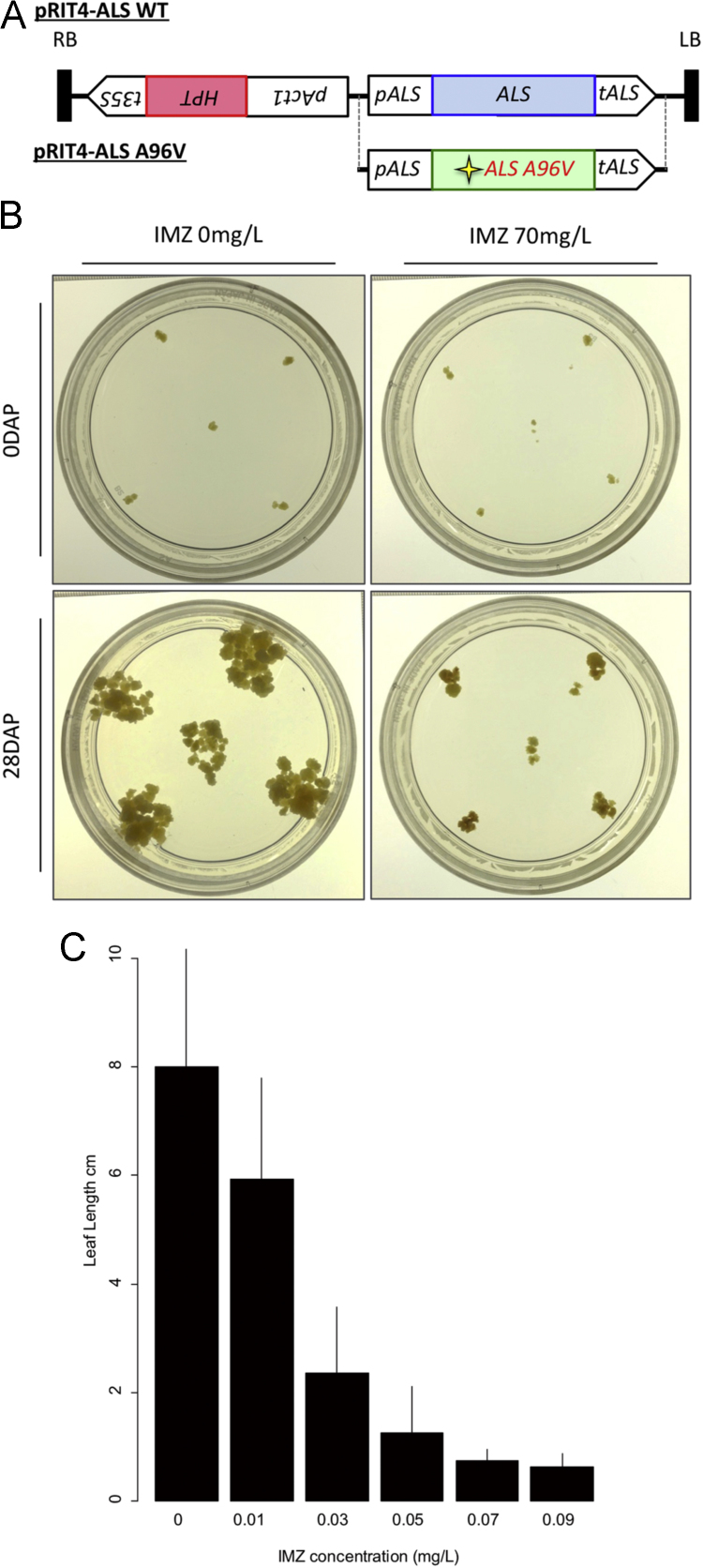
Imazamox tolerance-assay of rice calli and seedlings. (A) The T-DNA structures of the binary vectors used for Imazamox (IMZ) tolerance assay. The rice *acetolactate synthase* (*ALS*) gene was cloned and integrated into pRIT4 with its authentic promoter and terminator. The four-pointed star indicates the artificially induced nucleotide substitution leading to A96V mutation. *pAct1*, rice *Actin 1* promoter with its intron 1; *HPT*, *hygromycin phosphotransferase*; *t35S*, cauliflower mosaic virus *35S* terminator; RB, right border; LB, left border. (B) Effect of IMZ on proliferation of rice callus. Rice calli were cultured for 28 days on N6D medium supplemented with 0 and 70 mg/L IMZ. (C) Effect of IMZ on growth of rice seedlings. Rice seeds were germinated and grown for 10 days on 1/2MS medium supplemented with 0–0.09 mg/L IMZ under continuous light conditions. Mean lengths of leaf were indicated (*n* > 8). Bar = SD.

**Fig. 2:**
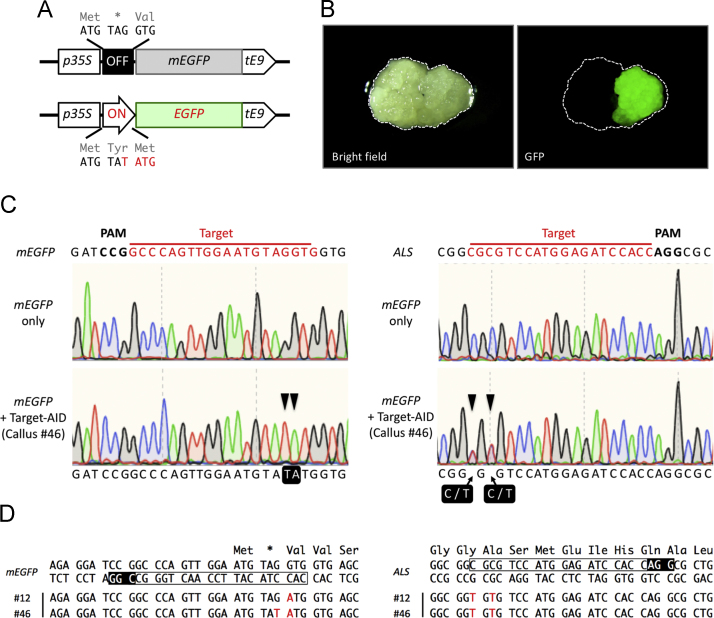
Simultaneous multiplex target editing by ALS-assisted Target-AID. (A) Schematic illustration of EGFP reporter assay. In *switch-mEGFP* reporter vector, single stop codon inserted immediately downstream of the initiation codon (Top). The switching module, TAG was altered to TAT by Target-AID to express EGFP (Bottom). (B) Fluorescence microscopic images of the rice callus. Expression of EGFP reporter was detected among double transformed callus lines carrying *mEGFP* and Target-AID. (C) Sequencing chromatograms showing the nucleotide substitutions by Target-AID in *switch-mEGFP* and *ALS* genes of calli carrying *switch-mEGFP* only (Top) and exhibiting EGFP expression and IMZ tolerance after introduced Target-AID vector (Bottom), respectively. Arrowheads with highlighted character indicate the mutation. (D) Sequence alignment of the mutations at *mEGFP* and *ALS* loci. Red letters indicate substituted nucleotides.

This article shows the optimization of an ALS-assisted screening strategy that facilitates more efficient targeted nucleotide substitutions in rice using Target-AID system. Optimal IMZ concentrations to inhibit rice callus proliferation and plant growth in tissue culture conditions were determined (Fig. 1B and C). The conferring of IMZ tolerance to rice calli by introducing *A96V* mutation in *ALS* was confirmed (Table 1). On the basis of these results, simultaneous multiplex gene editing with ALS-assisted Target-AID syrategy was demonstrated to introduce *A96V* mutation to endogenous *ALS* as well as restoration of *EGFP* (Fig. 2 B–D, Table 2).

**Table 1:** The efficiency of Imazamox selection of rice calli with *ALS A96V* mutation.

Vector	Number of callus lines		
	Hygromycin resistant	Imazamox tolerant	Frequency (%)
pRIT4-ALS WT	169	6	3.6
pRIT4-ALS A96V	263	261	99.2

**Table 2:** Number of rice calli carrying multiplex edited genes by Target-AID.

	Phenotype analysis	Sequencing analysis	
Analyzed	GFP-positive and Imazamox tolerant	*ALS A96V*	Recovered *EGFP*
124	3	2	2

## 2 Experimental design, materials, and methods

### 2.1 Evaluation of Imazamox tolerance of rice in tissue culture conditions

Effects of IMZ concentration in N6D media [3] on rice callus were evaluated. N6D and N6DSE-IMZ medium containing IMZ at gradual concentrations (30, 50, 70 mg/L) were used in this assay. The proliferation of rice callus was strictly inhibited by 70 mg/L IMZ (Fig. 1B).

Wild type rice *ALS* gene was cloned from genomic DNA by PCR amplification using the appropriate primers numbered as 1 and 2 in Table 3. The *ALS A96V* gene was synthesized via overlapping PCR procedure using the primers 1–6. The DNA sequence of the clones carrying *ALS* genes were confirmed using the primers 7–24. The cloned genes were installed to pRIT4, a derivative of binary vector pRIT3 [2] harboring a modified *HPT* gene [4]. The resultant vectors, pRIT4-ALS WT and pRIT4-ALS A96V (Fig. 1A) were introduced to rice calli by *Agrobacterium*-mediated transformation according to a previous report [5] using the plant media shown in Table 4. Transformed calli were selected on N6DSE-H40 medium over 3 weeks, then subcultured on N6DSE-H40IMZ70 medium to evaluate IMZ tolerance. After the selection over 2 months, 99.2% of calli introduced *ALS A96V* exhibited IMZ tolerance and proliferated on the media, whereas almost all the calli were sensitive to IMZ when introduced *WT ALS* (Table 1).

**Table 3:** List of oligonucleotides used in this study.

Serial number	Name	Sequence (5′--->3′)	Note
	pRIT4-ALS A96V		
1	ALS cloning-F	AGTCCCTGCAGGTTAATTAACTTGCGCTGCGTTTGTGCGGGTGCG	Construction of pRIT4-ALS vectors
2	ALS cloning-R	TGACGGTACCACTAGTTAGTAGTACCCAATAAGATCGACCGAAGAGA	
3	ALSA96V-F	CGGGCGGCGTGTCCATGGAGATCCACCAGGCGCTG	Generating *ALS A96V* variant by overlapping-PCR
4	ALSA96V-F2	GGCGTCAGCGACGTGTTCGCCTACCCGGGCGGCGTGTCCATGGAGATCCACCAGGCGCTG	
5	ALSA96V-R2	GAGCGCGTCAGCGCCTGGTGGATCTCCATGGACACGCCGCCCGGGTAGGCGAACACGTCG	
6	ALSA96V-R	TCCATGGACACGCCGCCCGGGTAGGCGAACACGTC	
	*ALS*		
7	pALS F-1	CATCCAATCGACTGACACGCGGGCCCAGAT	PCR and sequencing analysis of rice *ALS* gene
8	pALS R-1	GGTTTCTGGGTTTGGGCGAGAGGGAGAGAG	
9	pALS R-2	ATCTGGGCCCGCGTGTCAGTCGATTGGATG	
10	ALS F-1	CCGTAAGAACCACCAGCGACACCACGTCCT	
11	ALS F-2	GGAGACGCCCATAGTCGAGGTCACCCGCTC	
12	ALS F-3	CAGGGCCAAGATTGTGCACATTGACATTGA	
13	ALS F-4	CTTGGGCAACCCGGAATGTGAGAGCGAGAT	
14	ALS F-5	GGTGCTTCTGTGGCTAACCCAGGTGTCACA	
15	ALS R-1	TTAATACACAGTCCTGCCATCACCATCCAG	
16	ALS R-2	GTGTAATATTGTGCCGCCCACATCTGGTGC	
17	ALS R-3	CCAACCAGACGCAAGACCTGCTCAAGCAAT	
18	ALS R-4	TCGCCCTGCTCGTGGCGGAAGAGGTGGTTG	
19	ALS R-5	ATGTCCGCGCCCTTGCGGGGCTCGGCCGGC	
20	ALS R-6	GAGCGGGTGACCTCGACTATGGGCGTCTCC	
21	tALS F-1	GGCAAAGCACCAGCCCGGCCTATGTTTGAC	
22	tALS F-2	TCTATGCAATAGCTCTGAGTTAAGTGTTTC	
23	tALS R-1	GGAGAGTACTTCGTGTGATGACAGTTGAGC	
24	tALS R-2	CACATACAAACATCATAGGCATACCACTCT	
	*switch-mEGFP*		
25	SbfI-p35S-F	ATGCATCCTGCAGGCTCTAGAGGATCCCCCCTCAG	PCR and sequencing analysis of *mEGFP* gene on pRIT3-mEGFP
26	EGFP-NotI-R	AGCCGGGCGGCCGCTTTACTTGTACAGCTCGTCCA	
27	p35SF-1	CGCACAATCCCACTATCCTTCGCAAGACCC

**Table 4:** Media composition for plant tissue culture in this study.

Medium		N6D	N6DSE-H40	N6DSE-H40P50	N6DSE-IMZ	N6DSE-H40IMZ70	1/2MS	1/2MS-IMZ
Application		Callus proliferation	Selection of transgenic calli		Selection of *ALS A96V* calli		Germination	Selection of ALS A96V Plants
Basal medium		N6D	N6D	N6D	N6D		1/2MS	
Selective agents	Hygromycin	–	40 mg/L	40 mg/L	–	40 mg/L	–	–
	Paromomycin	–	–	50 mg/L	–	–	–	–
	Imazamox	–	–	–	30, 50, 70 mg/L	70 mg/L	–	0.01–30 mg/L
Gelling agents	Gelrite	4 g/L	4 g/L	–	4 g/L	4 g/L	4 g/L	4 g/L
	Agarose	–	–	8 g/L	–	–	–	–

The minimum effective concentration of IMZ on rice seedlings in aseptic conditions were determined as follows. Wild-type rice seeds were germinated and grown on 1/2MS media containing IMZ at concentration of 0.01, 0.03, 0.05, 0.07, 0.09, 0.1 and 0.25 mg/L (Table 4). The growth of the seedlings was analyzed by measuring their shoot length at 7 days after planting. As a result, seedling growth was remarkably suppressed by IMZ at 0.07 mg/L or higher concentration (Fig. 1C).

### 2.2 Multiplex editing of endogenous genes by Target-AID

To demonstrate the multiplex gene editing by Target-AID, the callus lines harboring pRIT3-mEGFP [2] were used in this experiment. Such calli were confirmed to carry dysfunctional *EGFP* (*switch-mEGFP*) containing a premature stop codon right after the initiation codon (Fig. 2A). A vector for Target-AID system expressing nCas9(D10A)-PmCDA1 with gRNAs corresponding to endogenous *ALS* and *mEGFP* was introduced by *Agrobacterium*-mediated transformation. After selection on N6DSE-H40P50 media, the double transformants were subcultured on N6DSE-IMZ70 medium over 2 months. As a result, 3 callus lines exhibiting IMZ tolerance and EGFP expression were obtained from 124 double transformants (Table 2, Fig. 2B). The targeted nucleotide substitutions were confirmed by direct DNA sequencing analysis using primers 10, 24 for endogenous *ALS* and 25–27 for *switch-mEGFP* (Table 3, Table 2 calli were found to harbor both of the desired mutations leading to *ALS A96V* and functional recovery of *EGFP* gene (Fig. 2A, C, D).
