# Supplementary material for: Herbicide tolerance-assisted multiplex targeted nucleotide substitution in rice
Source: Data Brief. 2018 Aug 30;20:1325–31. doi: 10.1016/j.dib.2018.08.124 (PMC6146504; doi:10.1016/j.dib.2018.08.124)
Supplement: Supplementary file 1 — Supporting information [file mmc1.docx]

**Conflict of interest**

The authors have no conflicts of interest directly relevant to the content of this article.
